# Intra-person multi-task learning method for chronic-disease prediction

**DOI:** 10.1038/s41598-023-28383-9

**Published:** 2023-01-19

**Authors:** Gihyeon Kim, Heeryung Lim, Yunsoo Kim, Oran Kwon, Jang-Hwan Choi

**Affiliations:** 1 Department of Computational Medicine, Graduate Program in System Health Science and Engineering, Ewha Womans University, Seoul, 03760 Korea; 2 Division of Mechanical and Biomedical Engineering, Graduate Program in System Health Science and Engineering, Ewha Womans University, Seoul, 03760 Korea; 3 Department of Nutritional Science and Food Management, Graduate Program in System Health Science and Engineering, Ewha Womans University, Seoul, 03760 Korea

**Keywords:** Computational biology and bioinformatics, Diseases, Health care

## Abstract

In the medical field, various clinical information has been accumulated to help clinicians provide personalized medicine and make better diagnoses. As chronic diseases share similar characteristics, it is possible to predict multiple chronic diseases using the accumulated data of each patient. Thus, we propose an intra-person multi-task learning framework that jointly predicts the status of correlated chronic diseases and improves the model performance. Because chronic diseases occur over a long period and are affected by various factors, we considered features related to each chronic disease and the temporal relationship of the time-series data for accurate prediction. The study was carried out in three stages: (1) data preprocessing and feature selection using bidirectional recurrent imputation for time series (BRITS) and the least absolute shrinkage and selection operator (LASSO); (2) a convolutional neural network and long short-term memory (CNN-LSTM) for single-task models; and (3) a novel intra-person multi-task learning CNN-LSTM framework developed to predict multiple chronic diseases simultaneously. Our multi-task learning method between correlated chronic diseases produced a more stable and accurate system than single-task models and other baseline recurrent networks. Furthermore, the proposed model was tested using different time steps to illustrate its flexibility and generalization across multiple time steps.

## Introduction

A chronic disease is a disease or condition that usually lasts for a long time. Some of the most common types are cancer, diabetes, and hypertension. Type 2 diabetes is a continuing metabolic disorder characterized by high blood glucose caused by insulin resistance of cells due to pancreatic dysfunction^1^. Hypertension is a condition in which blood flows through blood vessels at constant high pressure and is a major preventable risk factor for cardiovascular disease^2,3^. The prediction of chronic diseases plays a crucial role in health informatics. Early detection of chronic diseases and effective treatment in their early stages can prevent further complications and have always been helpful to patients. In addition, the burden of chronic diseases is widely accepted as one of the primary healthcare challenges. Maintaining clinical databases is becoming an important task in the medical field that is steadily being carried out^4^. Because clinical information about various disease-related features is collected for each patient, various diseases can be predicted together for a single person (intra-person) if the information is used correctly.

Various predictive models have been proposed recently to predict diseases using clinical time-series data. Several studies have used machine-learning models for early detection and prediction of chronic diseases based on an individual’s current condition, performing well in detecting diabetes^5^ and hypertension^6,7^. However, these methods cannot temporally relate time-series data, which is important for chronic diseases. Deep-learning techniques have demonstrated promising results on various prediction tasks because they provide a more efficient learning mechanism than conventional machine-learning and classification methods such as SVM and logistic regression. Long short-term memory (LSTM) models have performed well in analyzing time-series clinical data to forecast various diseases because they leverage the temporal relationships of the disease states in patients over time and capture the progression of the disease^8–11^. In Alakus et al.^12^, LSTM and convolutional neural networks with LSTM (CNN-LSTM) architectures successfully estimated the patients who were likely to be infected by COVID-19. The spatially and temporally deep CNN-LSTM architecture allowed it to be applied to various tasks involving sequential inputs and outputs^13,14^. The correlation between multiple prediction tasks for each disease was not explored in these single-task models.

Multi-task learning has been applied to leverage the relations between multiple tasks in various fields, such as the medical domain and natural language processing (NLP)^15–18^. Multi-task learning learns a set of related tasks concurrently to improve the overall performance of a deep-learning model. For instance, El-Sappagh et al.^16^ applied multi-task learning to use multimodal data and to solve classification and regression tasks related to Alzheimer’s disease. In addition, various multi-task learning schemes have been developed to prevent a model from overfitting to a particular task and to train multiple tasks successfully^17,18^. Chronic diseases share similar characteristics and are time series in nature. Consequently, considering chronic-disease data as a group of correlated diseases with a time-series structure is the intuitive solution for the chronic-disease prediction problem. Conventional multi-task learning methods may be a solution to this, but it has the disadvantage that the model may demonstrate confusion when performing the main task because all tasks are trained simultaneously with the same weights. Thus, we employed our multi-task learning strategy, periodic and central weighted learning (PCWL), to effectively learn multiple prediction tasks for different chronic diseases by periodically changing the focus while training a single model.

The main contributions of this paper are as follows:

- We first propose an *intra-person* multi-task deep-learning method that integrates multiple chronic-disease status prediction tasks, which can learn distinguishable features from an individual CNN layer and shared LSTM blocks.
- We introduce an effective multi-task learning strategy to train multiple prediction tasks and improve overall performance without overfitting an individual task during training in terms of accuracy, area under the curve (AUC), F1-score, and precision.
- Experimental results show that our proposed method can outperform single-task models and other recurrent network baselines, such as LSTM^19^, GRU^20^, and RNN^21^. The optimal weights for each task were also explored.

The rest of this paper is structured as follows. The related works for predicting chronic disease status are discussed in Section “Related works”. Section “Materials and methods” mainly describes the dataset and preprocessing, feature selection, the proposed multi-task prediction network and performance metrics. In Section “Results and discussion”, a series of results of our experiments are demonstrated. At last, Section “Conclusion” concluded our study.

## Related works

### Relation between chronic diseases

Patients with chronic diseases usually develop more than one disease simultaneously. García-Olmos et al.^22^ tried to identify comorbidity patterns in patients with chronic diseases by the number of comorbidities, age, and sex. Their results showed that 42% of the registered population had at least one chronic condition, and almost one-quarter of the population presented with multimorbidity. Four comorbidity patterns of 26 chronic health conditions were identified based on the level of the comorbidity burden. For instance, hypertension, lipid metabolism disorders, type 2 diabetes, and cardiac arrhythmia had an intermediate comorbidity rate. Teljeur et al.^23^ examined the nature of multimorbidity in a cohort of patients with type 2 diabetes. They demonstrated that 90% of patients had at least one additional chronic condition, with hypertension, heart disease, and arthritis having a high prevalence of multimorbidity. These studies support the correlation between multiple chronic diseases, but despite the prevalence of multimorbidity in the adult population, an intra-person multi-task learning scheme has not yet been attempted.

### Chronic-disease prediction

Several machine-learning techniques have been applied for chronic-disease prediction. Wu et al.^5^ proposed a novel model for predicting type 2 diabetes mellitus (T2DM) that applied an improved k-means algorithm for unsupervised clustering and the logistic regression algorithm for supervised classification. A one-year risk prediction model for hypertension was developed using a machine-learning algorithm, XGBoost, which generates an ensemble of classification trees^6^. The study showed that diseases such as type 2 diabetes, lipid disorders, and cardiovascular diseases (CVDs) are driving or associated features of incident essential hypertension. In Heo et al.’s study^7^, machine-learning-based hypertension prediction models were developed using logistic regression, naive Bayes, and decision trees.

Recently, various studies conducted in the medical domain have used recurrent neural networks to use the time relationships underlying the time-series data. LSTM networks have been used for classification tasks on clinical time-series data and outperformed other traditional classification methods, such as multilayer perceptron and ANNs. In Lipton et al.’s study^8^, multilabel classification of 128 diagnoses was trained based on the multivariate time-series data of intensive care units. It was the first study to evaluate the performance of LSTMs in recognizing patterns in multivariate time-series data. A recurrent neural network (RNN) with LSTM architecture was used in Reddy et al.’s study^11^ to predict the rehospitalization of lupus patients within 30 days by extracting the temporal relationships in the longitudinal electronic health record data. In addition, a combination of CNN and LSTM networks has been applied to leverage the spatial and temporal features for various tasks^12,16^. Alakus et al.^12^ developed and compared six deep-learning models to predict COVID-19 infection using laboratory data. The CNN-LSTM model obtained the best validation results using the train-test split approach to generate clear results in clinical applications^24^. The CNN-LSTM could extract both local and longitudinal features because the CNN functioned as an encoder and feature extractor and the LSTM acted as a decoder.

### Multi-task learning approach

Multi-task learning is the process of learning multiple related tasks simultaneously to improve the performance and generalizability of a model^25^. This learning strategy has been successfully developed and employed in various fields. Most studies are based on single-task models, which perform one clinical prediction task at a time. However, this approach does not reflect the reality of clinical decision-making, in which multiple tasks are performed simultaneously by clinical staff^15,26^. Therefore, Harutyunyan et al.^15^ proposed a multi-task framework that includes four different clinical prediction tasks using the MIMIC-III dataset: in-hospital mortality, physiologic decompensation, length of stay, and phenotype classification. The four prediction tasks were jointly learned simultaneously, and the results showed that channel-wise LSTMs and multi-task training work as a regularizer for almost all tasks. The model consisted of different baseline models for each task, and the overall loss was the weighted sum of the task-specific losses. In El-Sappagh et al.’s study^16^, a multimodal multi-task model based on a stacked CNN and bidirectional LSTM (BiLSTM) network was used to jointly learn a multiclass classification for Alzheimer’s disease and four cognitive-scores regression tasks. Deep features learned from each modality and the background data were fused by a set of shared dense layers, and task-specific learning was applied. Despite the attempt to use multi-task learning in the clinical field, most of the previous studies focused on the prediction of multiple tasks rather than the development of an effective multi-task learning strategy for time series data. Several multi-task learning strategies were developed for effective training and produced satisfactory results compared to single-task models and other multi-task variants. Yang et al.^18^ and Son et al.^17^ proposed a sequentially adaptive learning paradigm of multi-task learning weights for lung cancer prediction and multiple NLP tasks, respectively.

## Materials and methods

### Data

#### Dataset

**Figure 1:**
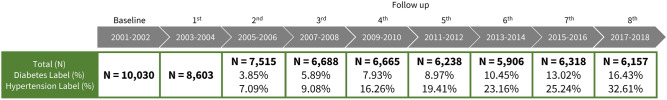
Flow diagram of baseline recruitment and follow-up for KoGES. Each time step was investigated at 2-year intervals, and each cell of the figure consists of the total number of patients in the original dataset at each time step (N) and the incidence rate (%) of 3379 and 2159 patients with diabetes and hypertension used as model inputs.

The Korean Genome and Epidemiology Study (KoGES)^27^ is an ongoing, prospective, large-cohort study conducted by the Korean government that started in 2001 (Fig. 1). All procedures performed in the study were in accordance with the ethical standards of the institutional research committee, and individual informed consent was acquired from the participants when the data were collected. It includes a biannual examination of lifestyle, biochemical profiles, and environmental factors related to the onset of common chronic diseases in Korean adults. We used the community-based cohort, a KoGES 16-year follow-up study of men and women aged 40–69 years living in Ansan, an urban city, and Anseong, a rural city. Detailed information on the study procedure has been described previously^28^. Of the original 10,030 participants, 3995 subjects remained enrolled after the eighth round of follow-up. In addition, subjects who had already been diagnosed with a disease or determined to have a disease at the baseline or first follow-up were excluded for diabetes or hypertension. This resulted in 3379 and 2159 subjects were enrolled in the diabetes and hypertension status prediction tasks, respectively. Diabetes mellitus was identified from a history of diagnosis, treatment, or insulin medication or according to the American Diabetes Association guidelines^29^: (1) a fasting glucose concentration ≥ 126 mg/dL, (2) a 2-h post glucose level ≥ 200 mg/dL in an oral glucose tolerance test, or (3) an HbA1C level ≥ 6.5%. Hypertension was defined based on a history of diagnosis, treatment, or antihypertensive medication or a current systolic pressure ≥ 140 mmHg or diastolic pressure ≥ 90 mmHg. The incidence rate for the eighth time step was 16.43% and 32.61% for diabetes and hypertension, respectively. In this study, only continuous variables were used in the data source. The study protocol was approved by the Institutional Review Board of Ewha Womans University (IRB No. 202106-0017-01). All experiments were performed in accordance with relevant guidelines and regulations. All participants provided written informed consent prior to enrollment, and their records were anonymously made prior to author’s access.

#### Dataset preprocessing

The KoGES data, like typical multivariate time-series data in health care, is missing some values that can cause inaccurate prediction results. To address this problem, we used bidirectional recurrent imputation for time series (BRITS)^30^ to fill in the missing values for our multivariate time-series data. The missing values are regarded as variables in the bidirectional RNN graph that can be effectively updated during backpropagation. BRITS jointly performs two tasks in one neural graph, the imputation and classification tasks, to alleviate the error-propagation problem from imputation to classification. Our dataset performed better than the PhysioNet Challenge 2012 dataset^31^, a healthcare dataset that was tested for the BRITS algorithm, outputting a lower mean absolute error (MAE) and mean relative error (MRE). We trained BRITS using an Adam optimizer with a learning rate of 1e−3 and batch size 64. This algorithm was used to prepare the model inputs before and after feature extraction.

#### Feature selection (LASSO)

The effective removal of irrelevant variables is crucial for good prediction performance and reliability of the model. To select the optimal set of features related to diabetes and hypertension, we used the least absolute shrinkage and selection operator (LASSO)^32^ regression. This algorithm uses the L1 absolute value penalty function to compress the coefficients of weakly correlated variables to 0 and retain more strongly relevant variables. As LASSO performs both feature selection and normalization, it improves the prediction accuracy while eliminating redundancy^33^. The penalty coefficient α was set as the significant number of digits that range from 1e−3 to 100 (α ∈ {n*e^k^|1 ≤ n ≤ 9, − 4 ≤ k ≤ 2}) and optimized based on grid search through fivefold cross-validation. The larger the penalty coefficient is, the greater the degree of sparseness and the greater the penalty intensity are^34^. Features with missing values of 80% or more in at least one time step were removed. Feature selection was performed after the missing values were processed through BRITS on features excluding those used in the diagnostic criteria for each disease. For diabetes and hypertension, the coefficient of 0.2 was selected as the optimal parameter, and 48 and 51 features were extracted from 56 and 54 features, respectively. Finally, the significant features for each time step are stacked to form the input of the prediction model. For instance, the model input for diabetes prediction is a three-dimensional tensor having a shape of [3379, 7, 48]. Because our proposed model consists of different task types for each batch through a balanced batch sampler, data of different sizes can be used together.

### Single-task models

#### Convolutional neural network

In the single-task models, a separate CNN subnetwork is used for each task. A 1D convolution is applied separately along the time dimension for every input vector and used to learn univariate time-series data. It expands every univariate time series to feature maps, which are abstract and informative features suitable for LSTM prediction^16^. Each value of the feature map is then fed into a nonlinear activation function, a rectified linear unit (ReLU). Following the activation function, dropout is applied to prevent overfitting.

Through feature selection, we discovered that most of the features were identified as common between diabetes and hypertension prediction. For instance, the features used as diagnostic criteria for diabetes were extracted as significant features for hypertension and vice versa. This also supports the fact that diabetes and hypertension, which are typical chronic diseases, are closely related. Thus, we determined that it was more appropriate for the proposed model to use both significant features for diabetes and hypertension, respectively. For both tasks, we applied one CNN layer of 50 filters with a 1 × 1 filter size to separately transform the time series of multiple tensors into a new feature space with the same dimension to give nonlinearity. This operation maintained the time step by applying one kernel to only one time step. Note that this operation can be used as an input layer for any neural architecture that simultaneously takes different time-series features as an input. Following the CNN layer, ReLU activation was applied to give nonlinearity, and dropout was used to prevent the model from overfitting. A 1 × 1 convolutional layer of 50 filters was used to minimize the computational cost and reduce the risk of losing information from the selected features.

#### Baseline models

*LSTM, GRU, RNN.* To leverage the temporal correlation in the time-series data, three commonly used model structures for dealing with time-series data were tested: the RNN, the gated recurrent unit (GRU), and the LSTM. These models can find temporal patterns in longitudinal data. An RNN is a sequential network that takes a predefined sequence as input. The output of a previous step is fed as input to the current step using the feedback network in a hidden state. Although an RNN works well with moderately sized data, it suffers from short-term memory and vanishing gradient problems as the number of data samples increases in size^32^. To solve this problem, LSTM was proposed to learn long-term and short-term dependencies in sequential data through memory cells. The LSTM cell’s gated and feedback architecture uses existing autocorrelation in the time series. The GRU is a variant of the LSTM, as it shares the gated architecture that controls sequential information in the cell. However, unlike LSTM, a GRU expands the information flow inside the gating unit without a separate memory cell.

The baseline models share the same architecture consisting of four stacked LSTM blocks, one fully connected layer, and a sigmoid layer for binary classification. Each LSTM block in the single-task model is structured with an LSTM layer, Tanh activation, and a dropout with a probability of 0.3. The output from the lower LSTM layer is forwarded to the upper layer. The CNN block performs a preprocessing step to learn local features and matches the feature dimensions of two tasks. The output of the CNN is then refined through the LSTM subnetwork to learn temporal relationships. To minimize data imbalance problems, a weighted random sampler was used to assign class weights inversely proportionally to the class frequencies. To compare the performance of single-task models, the stacked LSTM architecture of every model used the same output layer units of 50, 10, 10, and 10.

### Multi-task learning with PWL and CWL

Multi-task learning is a method that simultaneously learns related tasks in one model and improves the overall performance of several tasks. Through multi-task learning, a task with a relatively large amount of data can assist the learning of other tasks that lack labeled data and reduce the risk of overfitting^17^. We applied a multi-task learning scheme inspired by^17,18^ for our proposed model for effective training of the central task. Our proposed scheme PCWL consists of two stages: periodic weighted learning (PWL) and central weighted learning (CWL). PWL alternates the dominance of tasks throughout the training by changing the focus task every 20 iterations and multiplying the loss of auxiliary task by a relatively smaller number than the weight of the focus task. The cycle for changing the focus task was tested with 10, 20, and 40 iterations, and 20 iterations showed the most stable performance with the highest accuracy and AUC. CWL is applied in the final stages to improve the central task’s performance further by suppressing the loss functions on auxiliary tasks and thereby focusing on the central task.

**Figure 2:**
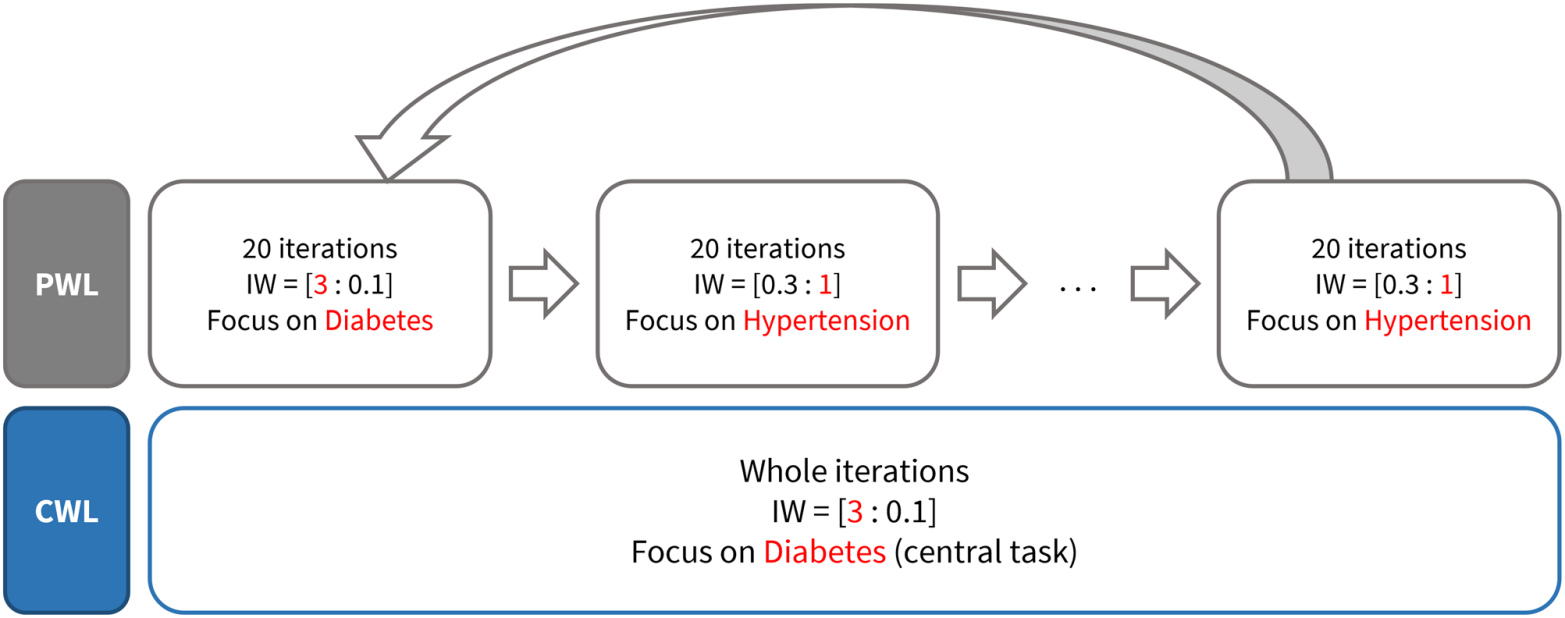
Example of multi-task learning applied with a periodic and central weighted learning (PCWL) scheme when the initial weight is fixed at [3:1]. In this example, diabetes and hypertension refer to each prediction task, and the central task is the diabetes prediction task. In the PWL stage, the focus task is changed every 20 iterations to give equal opportunity during training, and the weight of the auxiliary task is multiplied by 0.1. In the CWL stage, the central task is fixed as the focus task, and the weight of the auxiliary task is multiplied by 0.1. The red number and letters indicate the loss weight and type of the focus task, respectively.

In this paper, multi-task learning was applied to learn the diabetes and hypertension prediction tasks simultaneously. The central task refers to the task targeted to improve performance, and the remaining task is an auxiliary task. Therefore, the hypertension task is an auxiliary task when the diabetes prediction task is the central task. Moreover, a task focused on each iteration is denoted as the focus task. All tasks have fixed initial weights, and the weights for each task are adjusted during training. For each epoch, the PWL and CWL stages are applied alternately, and the model is aware of the type of prediction task. In the PWL stage, all tasks are dominantly trained with equal opportunity. Moreover, the focus task is sequentially selected every 20 iterations and is trained with a high weight, whereas the auxiliary task weights are multiplied by 0.1. Following the PWL stage, CWL is used to concentrate on the central task. In the CWL stage, the central task is intensively learned by multiplying the weight of the auxiliary task by 0.1 for every iteration. For both stages, the computed loss values of each task are multiplied by the adjusted task weights, and the overall loss is calculated as the weighted sum of the task-specific losses. Figure 2 shows an example of applying PWL and CWL to the diabetes and hypertension prediction task. In this example, the diabetes prediction task is the central task, and the initial weights are set to [3:1]. Our model exploits parameter sharing in the shared layers for multi-task learning, as illustrated in Fig. 2. This multi-task learning scheme works as a regularizer and prevents the model from overfitting to a specific task^33^.

### Proposed deep-learning model

**Figure 3:**
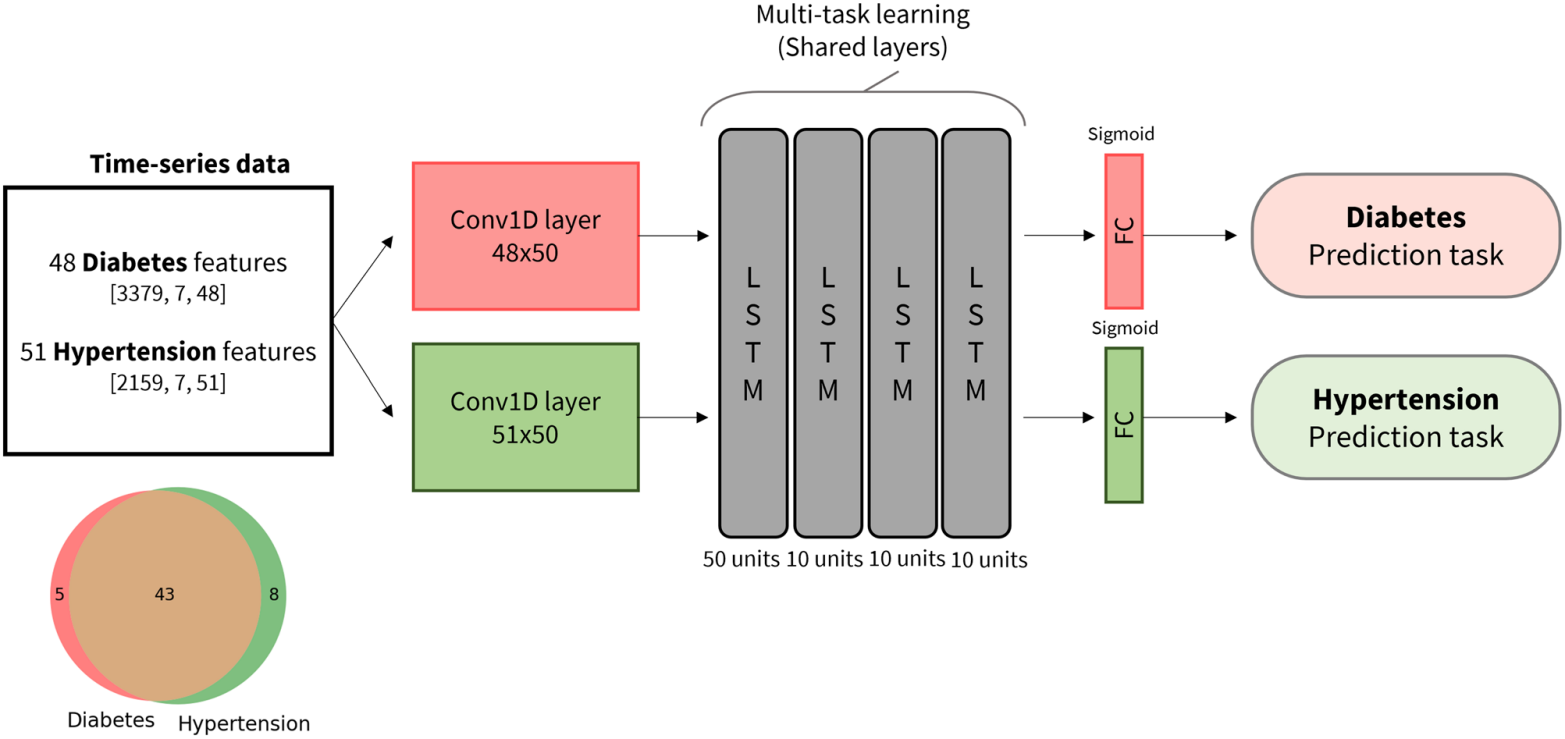
Schematic of the model architecture. A total of 48 and 51 clinical features were selected for diabetes and hypertension, respectively, with 43 features appearing to be commonly shared. First, the features of two different tasks were matched to the same dimension through the 1D convolution layer. Subsequently, the periodic weighted learning (PWL) and central weighted learning (CWL) schemes were applied on the shared long short-term memory (LSTM) blocks for multi-task learning. Lastly, the fully connected layer and sigmoid activation were used for the final prediction of the status of diseases. The input of the model has a data format in the form of [number of patients, time steps, features].

A schematic overview of the proposed network design is shown in Fig. 3. The proposed model introduces the concept of intra-person multi-task learning to predict the onset of typical chronic diseases, namely diabetes and hypertension, based on multivariate time-series data. Note that intra-person multi-task learning refers to a method in which a multi-tasking technique simultaneously learns several tasks in the shared layers of the model. First, data tracked over seven time steps for diabetes and hypertension are fed into the multi-task learning model. Learning local and temporal features is based on the CNN and shared LSTM subnetworks. As shown in Fig. 3, the model needs to prepare the time-series data consisting of significant features for both diseases. For time-series data, the features included data for seven time steps with two-year intervals, and the labels are based on whether the disease occurred in the eighth phase. Features significant for each disease are extracted by applying lasso regression to time-series data in which data preprocessing has been performed. Subsequently, deep features are concurrently learned using a stacked CNN-LSTM model. The CNN layer is applied for each task, and the stacked LSTM is shared by both tasks during training. Following the LSTM layers, the features learned from the shared layers are passed to task-specific layers for deeper feature learning. A task-specific layer consists of one fully connected layer and sigmoid activation to predict the occurrence of each disease at the eighth time step and to perform classification tasks.

### Performance metrics

For all algorithms, we split the diabetes and hypertension dataset of 3379 and 2159 cases into stratified datasets of 60% for training, 20% for validation, and 20% for testing. Stratification randomly distributes instances so that all datasets have similar ratios of different classes. The model was trained using the training set and optimized based on the validation set performance. The final scores are reported on the test set, which was unseen during model training. All experiments were repeated 10 times to prevent bias, and the final performance metrics were obtained by averaging the results. The performance was measured by the accuracy, AUC, F1-score, precision, and recall metrics. All models were trained with the Adam optimizer with a learning rate of 0.001 to minimize the weighted sum of task-specific losses. A sigmoid activation function with binary cross-entropy loss was used for both the diabetes and hypertension prediction classification tasks. The batch size and number of epochs were set to 32 and 300, respectively, for all experiments. To prevent overfitting, a dropout rate of 0.3 was applied after the CNN layer and every LSTM block. We used grid search to find the optimal layer units and model architecture. The proposed models were implemented using PyTorch as the back end.

Moreover, six different multi-task learning models (N-time-step models) were developed based on the clinical information accumulated for seven time steps. For instance, the two-time-step model predicts the eighth time step based on the last four years of progression. Then, we chose the best time-step model based on the prediction performance. Through these time-step models, we analyzed the evolution of the predictive performance over seven time steps and examined the stability and generality of our proposed model. The result of the N-time-step models was analyzed only for multi-task learning models with initial weights that performed best for each task.

## Results and discussion

Chronic diseases were predicted using various sources of clinical information, and they shared some features in common. Through LASSO feature selection, 48 and 51 features were selected for diabetes and hypertension prediction tasks, respectively, and 43 features were in common (Table 1). Detailed results and feature descriptions are described in Supplementary Table S1 online. All features except age were selected from the anthropometric and biochemistry measurements. Therefore, five and eight features were specifically selected for diabetes and hypertension, respectively. Among these features, those used in the diagnostic criteria of one disease and therefore removed in the feature extraction process were specifically extracted for the other disease.

**Table 1:** Summary of selected features for diabetes and hypertension.

	Characteristic
Diabetes only (5)	Protein, Sit-Left arm DBP, SBP, Sit-Right arm DBP, SBP
Common (43)	Age, ALT (SGPT), AST (SGOT), Fat-free mass, Body fat, Intracellular fluid, Body muscle mass, Body fat rate, BMI, Lie-DBP (1st), Lie-SBP (2nd), Sit-Left tactile SBP, Sit- Right tactile SBP, BUN, Creatinine, C-Reactive protein, Glucose (1-h OGTT), Hemoglobin, Hematocrit, HDL-Cholesterol, Height, Hip circumference (Pt, 2nd, 3rd), Insulin (fasting, 1-h OGTT, 2-h OGTT), Obesity degree, Urine (16), Platelet, Lie-pulse rate (1st), Blood-R.B.C, Subscapular (2nd, 3rd), Suprailiac (1st, 2nd, 3rd), Total cholesterol, Triglyceride, Waist circumference (1st), Blood-W.B.C, Weight
Hypertension only (8)	Mineral, Extracellular fluid, Lie-DBP (2nd), Fasting blood glucose, Glucose (2-h OGTT), Subscapular (1st), Waist circumference (2nd, 3rd)

A total of 48 and 51 features were selected for diabetes and hypertension, respectively, and 43 features were in common. The detailed description of the characteristics can be found in Supplementary Table S1 online. *BP* systolic blood pressure, *DBP* diastolic blood pressure.

Before combining the two prediction tasks, we tested the performance of each task alone and reported the average results. Three types of typical recurrent networks were tested as the baseline models for time-series data prediction. Table 2 shows the average results over seven time steps for three recurrent networks. LSTM was selected as the baseline model for both tasks because it had better AUC and recall metrics than the other recurrent networks, GRU and RNN. The hypertension prediction results are not as good as for diabetes, which can be considered natural as it concerns fewer samples with more balance between the labels. Initially, we used data augmentation techniques considering the labels of each time step to handle the label imbalance problem. However, this method did not work as expected because it showed an unstable loss in the baseline models.

**Table 2:** Comparison with baseline recurrent network models.

Task	Methods	Ace	AUC	F1	Precision	Recall
Diabetes	**LSTM**	85.37 ± 2.16	**93.57 ± 0.51**	65.61 ± 2.11	54.18 ± 5.45	**84.41 ± 5.78**
	GRU	**88.62 ± 1.1**	92.87 ± 0.38	**68.26 ± 1.68**	**63.45 ± 4.35**	74.41 ± 4.62
	RNN	88.46	92.35	67.22	62.99	72.07
Hypertension	**LSTM**	71.36 ± 7.18	**83.75 ± 0.75**	65.35 ± 3.33	55.85 ±7.59	**81.63 ± 10.2**
	GRU	**75.44 ± 2.41**	82.7 ± 0.6	**65.76 ± 0.86**	61.22 ± 5.29	72.4 ± 8.27
	RNN	67.82	62.58	4.14	**75**	2.13

The model with the best score for each disease is highlighted in bold.

To evaluate the performance and effectiveness of our proposed multi-task CNN-LSTM method, we tested and compared many schemes with different settings, including different model architectures and initial weights. Table 3 shows the results obtained from the single- and multi-task models for the prediction of the two chronic diseases, diabetes and hypertension. Before we applied multi-task learning, a single-task CNN-LSTM was tested to check the performance when the feature space of both tasks was set to the same dimension of 50. In diabetes prediction, the application of a CNN layer resulted in a slight decrease for most of the metrics. For hypertension prediction, there was a significant decrease in standard deviation and increase in accuracy, F1-score, and precision, 3.59%, 1.12%, 3.4%, respectively. The multi-task CNN-LSTM was tested using five sets of weights ([1:1], [3:1], [6:1], [9:1], [12:1], and [15:1]) to determine the optimal weight for each task. Overall, the multi-task CNN-LSTM achieved the best results. The results show that multi-task learning reduces the risk of overfitting and contributes to creating a more general and stable model than single-task models.

**Table 3:** Performance of single-task LSTM, convolutional neural network (CNN)-LSTM, and multi-task CNN-LSTM for the diabetes and hypertension prediction tasks.

Task	Methods		Acc	AUC	F1	Precision	Recall
Diabetes	LSTM		85.37 ± 2.16	93.57 ± 0.51	65.61 ± 2.11	54.18 ± 5.45	**84.41 ± 5.78**
	CNN-LSTM		85.03 ± 4.11	93.47 ± 0.41	64.99 ± 4.14	54.45 ± 7.92	82.79 ± 6.57
	Multi-task CNN-LSTM	**1:1**	**88.06 ± 1.31**	**93.89 ± 0.17**	**68.46 ± 1.47**	**61.01 ± 4.68**	78.64 ± 4.29
		3:1	87.14 ± 1.72	93.65 ± 0.36	67.39 ± 2.06	58.39 ± 5.09	80.45 ± 4.23
		6:1	87.05 ± 1.28	93.74 ± 0.22	67.32 ± 1.67	57.87 ± 3.99	80.99 ± 3.88
		9:1	86.80 ± 2.25	93.49 ± 0.37	67.06 ± 2.49	57.62 ± 5.20	81.17 ± 5.39
		12:1	86.71 ± 1.91	93.65 ± 0.33	66.94 ± 1.61	57.46 ± 5.53	81.44 ± 6.36
		15:1	87.64 ± 1.79	93.8 ± 0.25	67.81 ± 2.53	59.98 ± 5.77	78.73 ± 3.63
Hypertension	LSTM		71.36 ± 7.18	83.75 ± 0.75	65.35 ± 3.33	55.85 ± 7.59	**81.63 ± 10.2**
	CNN-LSTM		74.95 ± 1.65	83.07 ± 0.76	**66.47 ± 1.66**	59.25 ± 2.78	76.16 ± 5.69
	Multi-task CNN-LSTM	1:1	75.13 ± 4.13	83.60 ± 0.65	66.44 ± 1.43	60.65 ± 6.18	74.96 ± 7.64
		3:1	74.26 ± 4.88	83.32 ± 0.93	65.22 ± 1.87	60.53 ± 8.22	73.83 ± 11.94
		6:1	74.69 ± 3.45	83.56 ± 0.76	66.29 ± 1.88	59.42 ± 5.12	76.38 ± 7.17
		9:1	**75.87 ± 3.39**	83.39 ± 0.50	65.80 ± 2.42	**63.27 ± 8.45**	71.56 ± 11.93
		12: 1	75.09 ± 2.07	83.54 ± 0.74	65.90 ± 0.94	60.16 ± 4.55	73.82 ± 6.60
		**15: 1**	74.05 ± 2.18	**84.08 ± 0.66**	65.83 ± 1.59	58.36 ± 4.17	76.95 ± 9.49

We set the initial task weights of the central and auxiliary tasks to [1:1], [3:1], [6:1], [9:1], [12:1], and [15:1].

Best performance values are in bold.

The diabetes prediction task performed best for all metrics when the weight of 1 was applied, with an average accuracy of 88.06 ± 1.31%, AUC of 93.89 ± 0.17%, F1-score of 68.46 ± 1.47%, precision of 61.01 ± 4.68%, and recall of 78.64 ± 4.29%. Due to the significant imbalance in the diabetes dataset, increases in the AUC and F1 metrics have an important meaning. For hypertension, we selected a weight of 15 as the best initial weight because it produced the best AUC and F1-score metrics. It had an average accuracy of 74.05 ± 2.18%, AUC of 84.08 ± 0.66%, F1-score of 65.83 ± 1.59%, precision of 58.36 ± 4.17%, and recall of 76.95 ± 9.49%. Thus, it was confirmed that the initial weights we chose for the final model also followed the tendency of the initial weights according to the data size mentioned in a previous paper^17^. Because the hypertension task had a small data size, it performed better when a larger weight was applied than with diabetes. In addition, the hypertension task performance had a higher variance, and the weight application method was more effective.

Furthermore, Table 4 shows the results of the multi-task experiments we used to evaluate the effectiveness of our PWL and CWL strategy. The multi-task baseline was not applied with any adaptive weight policy, and the multi-task models with either PWL or CWL used the best initial weights for each task, which were [1:1] and [15:1] for diabetes and hypertension, respectively. The results show that all metric scores increased except the F1-score for the hypertension prediction when our multi-task learning strategy was applied. In particular, the results showed the best area under the curve (AUC) and recall, which are important evaluation metrics in clinical prediction tasks, when both strategies were applied. For diabetes prediction, the multi-task learning strategy showed improvement over the single-task model on all metrics except recall.

**Table 4:** Multi-task experiments of the PWL and CWL strategy.

Task	PWL	CWL	Acc	AUC	F1	Precision	Recall
Hypertension	Single-task model		74.95 ± 1.65	83.07 ± 0.76	**66.47 ± 1.66**	59.25 ± 2.78	76.16 ± 5.69
	0	X	**75.46 ± 4.13**	83.38 ± 1.04	66.3 ± 1.92	**61.83** ± 7.8	73.76 ± 9.73
	X	0	72.8 ± 6.15	83.23 ± 1.27	64.58 ± 2.81	58.22 ± 7.47	75.46 ± 11.85
	0	0	74.05 ± 2.18	**84.08 ± 0.66**	65.83 ± 1.59	58.36 ± 4.17	**76.95 ± 9.49**
Diabetes	Single-task model		85.03 ± 4.11	93.47 ± 0.41	64.99 ± 4.14	54.45 ± 7.92	**82.79 ± 6.57**
	0	X	**88.18 ± 1.15**	**93.94 ± 0.28**	**68.93 ± 1.78**	60.91 ± 3.46	79.64 ± 2.33
	X	0	87.63 ± 1.39	93.67 ± 0.23	67.79 ± 1.24	59.83 ± 4.57	79.01 ± 5.46
	0	0	88.06 ± 1.31	93.89 ± 0.17	68.46 ± 1.47	**61.01 ± 4.68**	78.64 ± 4.29

We set the initial task weights of the multi-task models to [1:1] and [15:1], for diabetes and hypertension prediction task, respectively.

Best performance values are in bold.

**Figure 4:**
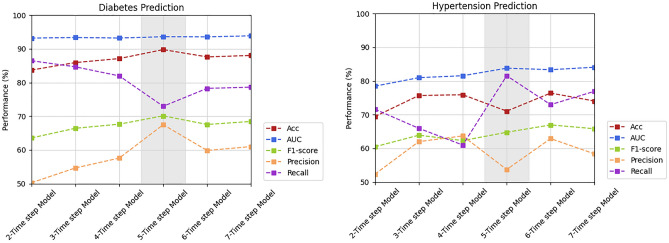
Graph illustrating the performance of N-time-step models for the diabetes and hypertension prediction tasks. For both tasks, the five-time-step model was considered the best because it achieved the best performance using the minimum time-series information.

Through Table 5 and Fig. 4, we can see the performance evolution of the multi-task CNN-LSTM models over time steps when the best initial weights were applied for each task. In diabetes prediction, it is clear that performance increased monotonically over time for almost all metrics. Nevertheless, the accuracy, F1-score, and precision metrics had peak performances in the five-time-step model. Although the best overall performance was obtained from the seven-time-step model, it is desirable to achieve good prediction with the lowest number of progression years. Thus, we considered the five-time-step model the most suitable for predicting diabetes, with accuracy of 89.79%, AUC of 93.62%, F1-score of 70.12%, precision of 67.5%, and recall of 72.97%. In hypertension prediction, an increase in accuracy, AUC, and F1-score is seen over time. As it is also desirable to leverage the least number of years that produces good performance, we selected the five-time-step model as the best, with accuracy of 71.06%, AUC of 83.81%, F1-score of 64.78%, precision of 53.73%, and recall of 81.56%.

**Table 5:** Performance of the proposed multi-task CNN-LSTM model for the diabetes and hypertension prediction tasks based on different time steps.

Task	Time steps	Acc	AUC	F1	Precision	Recall
Diabetes	7	88.06 ± 1.31	**93.89 ± 0.17**	68.46 ± 1.47	61.01 ± 4.68	78.64 ± 4.29
	6	87.63 ± 1.27	93.57 ± 0.31	67.57 ± 1.30	59.85 ± 4.44	78.28 ± 4.76
	**5**	**89.79**	93.62	**70.12**	**67.5**	72.97
	4	87.13	93.22	67.65	57.59	81.98
	3	85.94	93.38	66.43	54.65	84.68
	2	83.72	93.2	63.57	50.26	**86.48**
Hypertension	7	74.05 ± 2.18	**84.08 ± 0.66**	65.83 ± 1.59	58.36 ± 4.17	76.95 ± 9.49
	6	**76.41 ± 3.27**	83.36 ± 0.58	**66.96 ± 1.40**	62.90 ± 6.68	72.97 ± 6.98
	**5**	71.06	83.81	64.78	53.73	**81.56**
	4	75.92	81.56	62.31	**63.7**	60.99
	3	75.69	80.99	63.91	62	65.95
	2	69.44	78.46	60.47	52.33	71.63

Overall, the performance tended to decrease gradually as we used smaller numbers of time steps. For both tasks, using five time steps allowed the model to maintain performance with the least number of time steps. Using four time steps, the model performance degraded for some metrics: in the diabetes prediction task, the F1-score and precision metrics degraded, and there was a decrease in recall for the hypertension prediction task.

Best performance values are in bold.

The experiments using this model were conducted on diabetes and hypertension, the most representative diseases among chronic diseases in which clinical information was tracked up to the seventh time step. The results show that the multi-task technique helps reduce the standard deviation for almost all metrics compared to the single-task baseline models, thereby stabilizing the model. In addition, the proposed model can be generalized, as it can customize the initial weights and leverage all the significant features based on each task.

## Conclusion

In this paper, we propose an intra-person multi-task deep-learning model consisting of CNN and LSTM blocks to learn diabetes and hypertension status simultaneously. The CNN was used to extract local features from individual time series for each task, and the LSTM blocks were used to model the temporal variations and extract temporal features. The final multi-task CNN-LSTM model shares the LSTM blocks to apply multi-task learning with the optimal initial weights for each task. Through multi-task learning, the model can thoroughly learn all tasks while concentrating on the central task, thereby improving the overall performance. Experimental results of the proposed model demonstrated the effectiveness of multi-task learning in chronic-disease prediction using time-series data.

The proposed model can be applied to various predictive tasks in other medical domains because the model shows enhanced performance in time-series data with small data sizes. This approach shows that multiple chronic diseases can be predicted through various clinical information tracked from one patient because features selected as significant for one disease may also help predict other diseases. Further studies can investigate not only representative chronic diseases but also other diseases that can be learned concurrently to identify new relationships between diseases and improve the predictive performance of various diseases.

## Supplementary Information

Supplementary Table S1.

## Data Availability

The KoGES dataset used in this study has been anonymized and is open to the public by the Korea National Institute of Health (https://nih.go.kr/contents.es?mid=a50401010400).
