# Supplementary material for: Intra-person multi-task learning method for chronic-disease prediction
Source: Sci Rep. 2023 Jan 19;13:1069. doi: 10.1038/s41598-023-28383-9 (PMC9851106; doi:10.1038/s41598-023-28383-9)
Supplement: Supplementary file 1 — Supplementary Table S1. [file 41598_2023_28383_MOESM1_ESM.pdf]

Intra-person multi-task learning method for chronic-disease prediction

Gihyeon Kim, Heeryung Lim, Yunsoo Kim, Oran Kwon, Jang-Hwan Choi

Additional information

Supplementary information

	Characteristic	Description	LASSO coefficient	
			Diabetes	Hypertension
Common (43)	AGE	Age, years	0.005084	0.039385
	ALT_ORI	ALT (SGPT), IU/L	0.007943	-0.00063
	AST_ORI	AST (SGOT), IU/L	-0.007553	-0.000058
	BDCEXFT	Fat-free mass, kg	0.005135	0.000812
	BDCFT	Body fat, kg	0.102842	0.000048
	BDCINWT	Intracellular fluid, L	0.042784	0.003613
	BDCMSC	Body muscle mass, kg	0.000895	0.032531
	BDCWT	Body water, L	0.013967	0.033259
	BDFTR	Body fat rate, %	-0.016367	0.032806
	BMI	BMI, kg/m^2	-0.056062	0.004808
	BPLIE1D	Lie - DBP (1st), mmHg	-0.001038	0.010724
	BPLIE2S	Lie - SBP (2nd), mmHg	0.006522	0.027342
	BPSITL	Sit - Left tactile SBP, mmHg	-0.008607	0.022093
	BPSITR	Sit - Right tactile SBP, mmHg	0.000581	0.019701
	BUN_ORI	BUN, mg/dL	-0.018122	0.007663
	CREATININE_ORI	Creatinine, mg/dL	0.599667	0.139149
	CRP	C-Reactive protein, mg/dL	0.002122	0.006536
	GLU60_ORI	Glucose (1-h OGTT), mg/dL	0.032466	0.00473
	HB	Hemoglobin, g/dL	-0.110856	-0.032181
	HCT	Hematocrit, %	-0.006471	-0.013708
	HDL_ORI	HDL-Cholesterol, mg/dL	-0.011587	0.002796
	HEIGHT	Height, cm	-0.045274	-0.114626
	HIP1	Hip circumference (1st), cm	-0.001803	-0.001281
	HIP2	Hip circumference (2nd), cm	-0.00047	-0.009004
	HIP3	Hip circumference (3rd), cm	-0.000205	-0.000882
	INS0	Insulin (fasting), μIU/mℓ	0.048585	0.021947
	INS120	Insulin (2-h OGTT), μIU/mℓ	0.003073	0.001338
	INS60	Insulin (1-h OGTT), μIU/mℓ	-0.014758	-0.001708
	OBDG	Obesity degree, %	-0.010646	-0.036284
	PH_U	Urine (16) - pH	-0.019102	0.009046
	PLAT	Platelet, 10^3/μL	0.000999	0.001099
	PLLIE1	Lie - pulse rate (1st), bpm	-0.001344	0.00704
	RBC	Blood - R.B.C 10^6/μL	0.05389	0.06034
	SUB2	Subscapular (2nd), mm	-0.031222	-0.020491
	SUB3	Subscapular (3rd), mm	0.053911	0.034426
	SUP1	Suprailiac (1st), mm	-0.023569	0.011721
	SUP2	Suprailiac (2nd), mm	-0.011968	-0.01558
	SUP3	Suprailiac (3rd), mm	0.014935	-0.00999
	TCHL_ORI	Total cholesterol, mg/dL	-0.003325	-0.003997
	TG_ORI	Triglyceride, mg/dL	0.002108	0.000422
	WAIST1	Waist circumference (1st), cm	0.002054	0.009625
	WBC	Blood - W.B.C, 10^3/μL	0.041984	-0.018049
	WEIGHT	Weight, kg	0.013548	0.051384
Diabetes only (5)	BDCPRT	Body Protein, kg	-0.046126	-
	BPSIT1LD	Sit - Left arm DBP, mmHg	0.001151	-
	BPSIT1LS	Sit - Left arm SBP, mmHg	0.012864	-
	BPSIT1RD	Sit - Right arm DBP, mmHg	-0.014911	-
	BPSIT1RS	Sit - Right arm SBP, mmHg	-0.002204	-
Hypertension only (8)	BDCMN	Mineral, kg	-	-0.167453
	BDCOTWT	Extracellular fluid, L	-	0.130295
	BPLIE2D	Lie - DBP (2nd), mmHg	-	0.018817
	GLU0_ORI	Fasting blood glucose, mg/dL	-	-0.003225
	GLU120_ORI	Glucose (2-h OGTT), mg/dL	-	-0.001644
	SUB1	Subscapular (1st), mm	-	0.010817
	WAIST2	Waist circumference (2nd), cm	-	0.008232
	WAIST3	Waist circumference (3rd), cm	-	0.002546

Supplementary Table S1. Detailed description of the total features selected by LASSO regression. The characteristic names come from the open codebook provided by the KoGES dataset. Features can be divided into

common features and individual features related to diabetes and hypertension. SBP, systolic blood pressure; DBP, diastolic blood pressure;
